# Cellular and Molecular Effects of Magnetic Fields

**DOI:** 10.3390/ijms25168973

**Published:** 2024-08-17

**Authors:** Maciej Tota, Laura Jonderko, Julia Witek, Vitalij Novickij, Julita Kulbacka

**Affiliations:** 1 Student Research Group № K148, Faculty of Medicine, Wroclaw Medical University, 50-367 Wroclaw, Poland; maciej.tota@student.umw.edu.pl; 2 Student Research Group № K148, Faculty of Pharmacy, Wroclaw Medical University, 50-367 Wroclaw, Poland; laura.jonderko@student.umw.edu.pl (L.J.); 341725@uwr.edu.pl (J.W.); 3 Institute of High Magnetic Fields, Vilnius Gediminas Technical University, LT-03227 Vilnius, Lithuania; vitalij.novickij@vilniustech.lt; 4 Department of Immunology, State Research Institute Centre for Innovative Medicine, Santariškių 5, LT-08410 Vilnius, Lithuania; 5 Department of Molecular and Cellular Biology, Faculty of Pharmacy, Wroclaw Medical University, 50-367 Wrocław, Poland

**Keywords:** magnetic fields, cellular, molecular, biological, neurological, therapy, nanoparticles

## Abstract

Recently, magnetic fields (MFs) have received major attention due to their potential therapeutic applications and biological effects. This review provides a comprehensive analysis of the cellular and molecular impacts of MFs, with a focus on both in vitro and in vivo studies. We investigate the mechanisms by which MFs influence cell behavior, including modifications in gene expression, protein synthesis, and cellular signaling pathways. The interaction of MFs with cellular components such as ion channels, membranes, and the cytoskeleton is analyzed, along with their effects on cellular processes like proliferation, differentiation, and apoptosis. Molecular insights are offered into how MFs modulate oxidative stress and inflammatory responses, which are pivotal in various pathological conditions. Furthermore, we explore the therapeutic potential of MFs in regenerative medicine, cancer treatment, and neurodegenerative diseases. By synthesizing current findings, this article aims to elucidate the complex bioeffects of MFs, thereby facilitating their optimized application in medical and biotechnological fields.

## 1. Introduction

### 1.1. Magnetic Field Parameters

The biological impacts of magnetic fields (MFs) can be directly affected by various factors. Depending on whether there is a change in magnetic intensity over time, MFs can be categorized as static magnetic fields (SMF) or dynamic/time-varying magnetic fields, further classified based on their frequency. The tesla (symbol: T) represents the unit of MF intensity within the International System of Units (SI). Depending on the strength of the MF, there are weak (<1 mT), moderate (1 mT to 1 T), high (1–20 T), and ultra-high (20 T and above) MFs [1]. Understanding these thresholds is crucial for harnessing the therapeutic potential of magnetic fields in medicine and ensuring safety in environments where humans are exposed to such fields (Table 1). Further research is needed to elucidate the mechanisms behind these effects and to optimize MF parameters for specific therapeutic applications.

### 1.2. Magnetic Fields and Magnetosensitivity of Biological Systems

Biological molecules interact with different spectra of MFs, and their response depends on the intensity of these fields [17]. The impact of MFs on biological molecules is caused by their magnetic properties, which originate from electrons’ magnetic dipole moment and spin magnetic moment [18]. Due to the scale of magnetic property strength, substances are divided into three groups: ferromagnetics (e.g., Fe, Ni, Co, CrO_2_), paramagnetics (e.g., O_2_, Cu^2+^, Fe^3+^, free radicals), and diamagnetics (e.g., NaCl, KCl), in which ferromagnetics have the strongest magnetic properties, while diamagnetics have the weakest [19].

Many biological molecules are referred to as paramagnetics or diamagnetics. Research has delivered proof about the diamagnetic properties of cellular organelles like the cytoskeleton (microtubules), cell membrane lipids, DNA strands, and some membrane receptors [1]. It is proven that the structure of the molecule is associated with its magnetic properties; therefore, the diamagnetic properties of the mentioned biomolecules result from their α-helical structure [20]. These physical quantities cause the magnetosensitivity of biological systems [21].

### 1.3. Geomagnetic Fields (GMFs)

The major impact on biological systems and their compounds is Earth’s weak MFs (25–65 μT), also referred to as geomagnetic fields (GMFs), whose sources are Earth’s core and magnetic poles at the external Earth’s surface [22]. It is known that GMFs play an essential role in life evolution on Earth because they stabilize environmental components like the development of the ozone layer or temperature reduction [23]. However, the GMFs also have a fundamental role in cells, tissues, and organisms’ functionality [24]. Due to the evolution under Earth’s GMF conditions, a lack of it causes various dysfunctions in biological systems; this reduced GMF is referred to as a hypomagnetic field (HMF) [25].

It is important to point out that HMFs have an impact on various organisms: animals, plants, and bacteria [26]. It is proven that HMF inhibits plant flowering, affecting different gene expressions dependent on light spectra [27]. Emerging evidence suggests that HMF causes embryonic abnormalities and has an impact on animal reproduction, including mammals, amphibians, and arthropods [28]. HMF affects cellular homeostasis by disturbing many cellular processes, for example, promoting ROS (reactive oxygen species) release, leading to metabolic disruptions [29]. Emerging evidence suggests that HMF leads to cell growth rate reduction. For example, Martino et al. found that HMF conditions inhibit fibrosarcoma and colorectal cancer cell proliferation [30]. Evidence suggests that HMF also causes various adverse effects on human health. HMFs disturb circadian rhythms due to affection melatonin and norepinephrine release. These two compounds participate in the biological clock adaptation [31]. Homeostatic alterations in biological systems are caused by changes in MF intensity, e.g., properties of biological membranes like membrane potential, changes in enzyme activities, progression of circadian rhythm, reactive oxygen species production, DNA replication, chromatin condensation, cell morphology, neuronal activities, gene expression, cellular organelle functioning, ion homeostasis, oxygen transport chain progression, and cell cycle promotion [21,24,32].

Moreover, GMFs are embraced by many species, like animals, i.e., birds or magnetotactic bacteria, to navigate [33]. Magnetotactic bacteria can sense GMF due to specific organelle magnetosomes [34]. Magnetosomes enable bacterial cells to move along an external geomagnetic field [35]. This phenomenon is referred to as magnetoreception, and there is a hypothesis that flavoprotein cryptochrome (CRY2) is involved in geomagnetic field sensing. Interestingly, humans are unable to sense geomagnetic fields despite the fact that they also express the CRY2 protein [36]. Foley et al. delivered experimental proof that human CRY2, expressed in *Drosophila melanogaster* cells, could serve as a light-dependent magnetosensor [37]. However, in animal or human tissues, biogenic magnetic nanoparticles, especially magnetite, interact with MF [38]. The genesis of these particles is not fully understood but could be a reason for iron overload due to mitochondria dysfunction and age, diseases, symbiotic and pathogenic microorganisms, genetically programmed biosynthesis, and environmental conditions [39].

The aim of this review is to investigate the cellular and molecular effects of MFs on biological systems. By examining various cellular responses and molecular mechanisms, we seek to elucidate how exposure to MFs influences cellular differentiation, proliferation, cell morphology and motility, gene expression, DNA damage, genotoxicity, protein function, and signal transduction pathways. Moreover, this study aims to provide a comprehensive understanding of the biological impacts of MFs, which could have significant implications in neurology, radiology, cardiology, and oncology.

## 2. Biological Properties of Membranes

**Figure 1:**
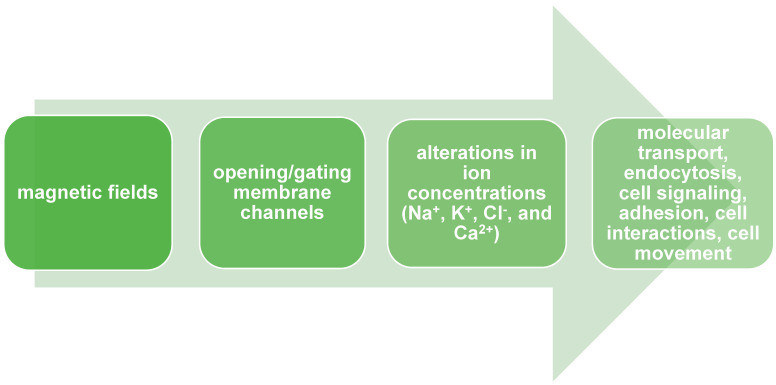
Magnetic fields modulate biological properties of cells by opening or gating membrane channels.

Cellular membranes are characterized by electrical properties like membrane potential [40]. The membrane potential changes through a cell cycle progression, leading to depolarization or hyperpolarization of the cell membrane [41]. Membrane potential is generated by ion flux balance, especially Na^+^, K^+^, Cl^−^, and Ca^2+^, across cell membranes [42]. There is experimental evidence that MF impacts the opening/gating of membrane channels and activation/deactivation of ion pumps or specific carrier proteins [43]. For instance, Alaya et al. showed that spatiotemporal MF (17–70 mT) induces an influx of sodium ions by opening voltage-gated sodium channels (VGSCs) [44]. Yan and colleagues delivered evidence that ELF-MF (50 Hz, 20 mT) increases Na+ and K+ extracellular concentrations in human mesenchymal stem cells. The authors also suggested that the phenomenon could be a result of opening ion channels induced by MF [45]. Thus, MFs impact membrane potential changes, which could reprogram cell fate [46]. Zablotskii and colleagues, in their research, show that a high gradient magnetic field (HGMF), which has 106–107 Tm-1, could cause cell membrane depolarization or hyperpolarization. The researchers pointed out which cellular mechanisms led to these phenomena: opening or gating magnetosensitive ion channels, an alteration in ions flux balance, mechanical stress in the membrane, and migration of receptor proteins [46]. These mechanisms could be cellular responses to alteration in homeostasis with HGMF. These changes play a crucial role in numerous cellular processes, e.g., molecular transport, endocytosis, cell signaling, cell dividing, adhesion, apoptosis, cell interactions, shaping, and cell movement [47] Figure 1 There is experimental proof that extremely low-frequency (ELF-MF) magnetic fields interact with Ca^2+^ channels, leading to increased Ca^2+^ efflux [48]. Wang and colleagues, in their research, have also shown a connection between static MF exposure and increased Ca^2+^ efflux in the PC12 cell line [49]. Gorobets and colleagues showed that time-varying MF decreased intracellular Ca^2+^ concentrations because of alterations in ion channel activity. They suggested that the occurrence of biogenic and non-biogenic magnetic nanoparticles in cell membranes led to shear stress and modulated the activity of Ca^2+^ channels [23]. There is a hypothesis that describes MF’s impact on ion channels. MF may cause the reorganization of lipids in lipid rafts, which modulate protein activity [1].

On the other hand, MF could increase Ca^2+^ concentrations in cells. The research of Bekhite et al. delivered results that proved the ability of MF (1 mT) to stimulate Ca^2+^ influx [50]. Except for membrane potential generation, Ca^2+^ participates in many cellular signaling pathways as secondary messengers, for example, specific gene expression, mitochondrial permeability transition, ROS production, cell proliferation, stress response, cell movement, and synaptic activity [51]. Wu and colleagues delivered experimental evidence that static MF regulates T-type Ca^2+^ channels in human mesenchymal stem cells, resulting in cell membrane depolarization. They found that the regulation led to the activation of an MAPK signaling pathway and thus increased cell proliferation [52].

MF alters not only the membrane potential but also other membrane properties, like permeability, by opening membrane pores [53]. There is accumulating data proving that pulsed MF increases cell membrane permeability and cellular transport [54]. Novickij and colleagues found that a pulsed electromagnetic field (5.5 T) increased cell membrane permeabilization in the CHO-K1 cell line [55]. Interestingly, permeabilization did not lead to cell death. The reversibility of permeabilization utilizing MF was also demonstrated by Novickij et al. [56]. 

It is important to point out the fact that MF forces induce changes in the membranes of cellular organelles too, like mitochondria. The modeling by Ye et al. delivered information about alterations in mitochondrial physiology during time-varying magnetic stimulations. They found that MF-induced membrane potential alterations in mitochondria are the same as those in cell membrane potential [57]. The accumulated evidence suggests that MF increases mitochondrial permeability transition [58]. Feng and colleagues proved that ELF-MF (50 Hz and 0.4 mT) increased mitochondrial membrane permeability by modulating ROS/GSK-3β signalization. Released ROS leads to GSK-3β dephosphorylation and thus opens a mitochondrial permeability transition pore (mPTP) [59]. Evidence collected by Franco-Obregón suggests that ELF-EMF has an impact on the mitochondrial stress response process—mitohormesis [60].

## 3. Cell Morphology and Motility

The cytoskeleton is connected to cellular mechanics. It enables the cell to maintain its shape and is also involved in cell division, movement, adhesion, and intracellular trafficking [61]. Data collected by Gartzke et al. suggest that weak MF has an impact on actin filaments of microvilli, especially ion conduction along them [62]. Literature suggests that GMF plays an essential role in cytoskeleton assembly, and its absence leads to cell reshaping and the inhibition of cellular movement. Mo and colleagues delivered experimental proof that a lack of GMF resulted in a reduction in F-actin in neuroblastoma cells. A decreased concentration of F-actin led to disorganization of the cytoskeleton and inhibited cell migration and adhesion [63]. Zablotskii and colleagues showed that the MF gradient induces a cell’s paramagnetic properties and thus leads to cell adhesion and migration in that gradient [64]. As an adaptation to changes in MF intensity, the cell remodels the cytoskeleton and consequently reshapes [65]. Also, changes in intracellular Ca^2+^ concentration have an impact on cytoskeleton reorganization, especially microtubules and microfilaments, which leads to cell shape alteration [20]. Zhang and colleagues found an ultra-high SMF (27 T) impact on cell cytoskeleton reorganization, especially mitotic spindle reorientation and morphology [66]. The mitotic spindle plays an important role in cell division due to chromosome segregation during the mitotic and meiotic processes [67]. The mitotic spindle is built by polarized microtubules, which provide a proper orientation [68], and the influence of SMF results in reorientation. They found that the changes in the mitotic spindle had consequences for chromatin and microtubule alteration, too [66]. Alterations in cytoskeleton rearrangement could affect cellular morphology, such as cell shape—from round to more irregular and elongated—or loss of adhesion [69]. Evidence shows that exposure to SMF causes cell membrane morphological modifications, like blebs or microvilli. Changes in cellular membrane morphology lead to alterations in membrane dynamics, adhesion, and protein interaction [70]. Chionna et al. delivered evidence that SMF causes shape-changing and membrane microvilli alterations in lymphocytes and monocytes [71]. Information accumulated by Albuquerque et al. showed a connection between MF-induced, altered calcium ion metabolism and cytoskeleton rearrangement. 

In some cases, intracellular growth of Ca^2+^ results in the reorganization of microtubules and microfilaments, consequently leading to cell reshaping [20]. Ayala and colleagues proved that spatiotemporal MF (17–70 mT) causes intracellular calcium ion concentration growth and, thus, induction of actin polymerization in skeletal muscle cells [44]. Experimental evidence reports HGMF-induced F-actin distribution changes [72]. Wosik and colleagues suggest that MF affects cytoskeleton rearrangement in macrophages. The basis of the rearrangement is alterations in the polymerization of actin filaments, which lead to cell reshaping and elongation [73]. Similar conclusions were made by Perez et al. about F-actin polymerization changes in human mesenchymal stem cells [72]. In addition, it was suggested that MF affects cellular adhesion, leading to focal adhesion by modulating vinculin redistribution [73]. Besides cell adhesion, there is evidence that MF affects cell migration. Ji and colleagues delivered proof that low-frequency rotating MF (0.1 and 0.4 T), through F-actin modulation, inhibits breast cancer cell migration [74].

## 4. Cellular Differentiation and Proliferation

**Figure 2:**
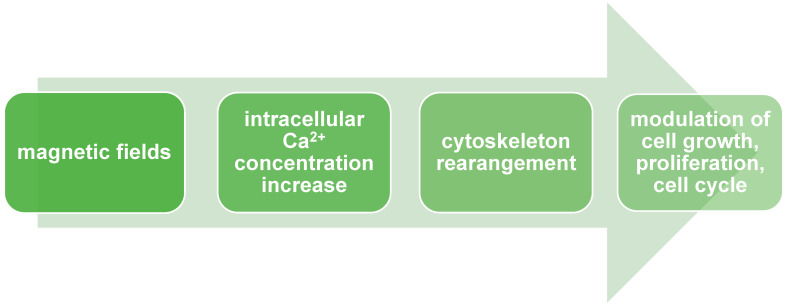
Magnetic fields modulate cell growth, proliferation, and cell cycle via Ca^2+^ signaling.

We mentioned above that MF alters ion flux balance and intracellular ion concentration. These alterations lead to changes in the cell’s membrane potential and result in the modulation of cell growth, proliferation, and the cell cycle. Additionally, the impact of MF on alterations in Ca^2+^ concentration levels was also described. Changes in Ca^2+^ intracellular concentrations have an essential impact on cell fate because they participate in various signaling pathways involved in crucial physiological processes, e.g., cell proliferation, differentiation, cell cycle, circadian rhythm, muscle contraction, and signal transduction in the nervous system [15]. Increased Ca^2+^ intracellular concentration affects cytoskeleton rearrangement (Figure 2). Cytoskeleton components (like actin and integrin) play an essential role in cellular differentiation through genome organization and the regulation of exact gene expression. Interactions between Ca^2+^ and the cytoskeleton also result in changes in the proliferation rate. What is important to point out is that SMF exposure results in different proliferation rate responses depending on cell type. 

Moreover, different directions of MF also have an impact on cellular mechanics, which was noticed by Tian and colleagues [75]. The positive effects of SMF exposure on the proliferation rate of mesenchymal stem cells (MSCs) were noticed by Wu et al. The authors detected alterations in cellular membrane potential via Ca^2+^ flux balance changes, which resulted in the depolarization of the cell membrane. The investigation pointed to T-type Ca^2+^ voltage-gated channels as MF sensors [52]. Interestingly, the alteration in intracellular Ca^2+^ concentration affects cell migration. Zhang and colleagues used ELF-EMF to promote human bone marrow-derived mesenchymal stem cell migration. They found that the migration process was driven by intracellular Ca^2+^ levels [76]. There is accumulated evidence that MF alters stem cell properties like proliferation or differentiation. One of the potential applications of stem cells is wound healing [77]. Moreover, it also proves an anti-inflammatory response as a result of MF exposure.

On the other hand, Kim and colleagues found that ELF-EMF could promote the pro-inflammatory response of macrophage cell lines [78]. There are experimental proofs that MFs have an impact on signaling pathways involved in cell proliferation. As a great example, serve Lew and colleagues’ results. The authors suggested that SMF (0.4 T) enhanced the proliferation rate of dental pulp mesenchymal stem cells by activating the p58/MAPK signaling pathway. Lew and colleagues also noticed increased intercellular Ca^2+^ concentrations [79]. Marędziak et al. also found that interaction between MF and activation of signaling pathways results in enhanced proliferation. SMF (0.5 T) had an impact on the activation of the PI3K/Akt pathway and thus led to enhanced proliferation in human adipose-derived mesenchymal stromal stem cells [80]. There are associations between PEMF exposure and promoting osteogenesis and chondrogenesis of mesenchymal stem cells [81]. A high range of MF could serve as a regulator of differentiation and proliferation processes. Inhibition of the osteoclastic bone resorption process was detected during high static MF (16 T) exposure [82].

Moreover, PEMF stimulates the anti-inflammatory response of mesenchymal stem cells. Varani et al. pointed out that PEMF’s therapeutic effects could serve as applications in cartilage and bone treatment [83]. Luoa et al. observed alterations in periodontal ligament stem cell physiology after SMF (320 mT) exposure. Interestingly, the cell proliferation rate increased, and the cells were characterized by osteogenic differentiation markers. The authors found that these changes were induced by the modulation of Akt signaling pathways [84].

It is important to point out that MF also has an anti-proliferative impact on cancer cells. Delivered data suggest MF exposure results in a cell-type-dependent manner. These results also suggest the usage of MF as a non-invasive and targeted approach in cancer treatment [85]. For example, Zhang et al. delivered data that SMF (1 T) exposure results in a decreased proliferation rate of a nasopharyngeal carcinoma cell line. Potentially, these alterations were caused by inhibition of the Akt/mTOR signaling pathway [86]. MF could affect the biological membrane physiology of cancer cells and lead to a decrease in growth and viability. Ashdown and colleagues observed disruptions in the human lung cancer cell line after PMF (20 mT) exposure; in comparison, normal cells were insensitive to PMF [87]. MFs alter the membrane potential of cancer cells, leading to the depolarization of suspended cells and the hyperpolarization of adherent cell lines [87]. Sun and colleagues suggested that during MF exposure, cells were releasing substances that inhibited cancer cell line growth but did not affect non-tumorous cells [88]. MF exposure also affects cancer cell migration. Furthermore, Fan et al. suggested a mechanism in which SMF (150 mT) inhibits telomerase activity, thus shortening telomeres and leading to a decreased proliferation rate of breast cancer cells [89]. There are possibilities to utilize MF in cancer treatment and combine it with other therapies.

## 5. Potential Genotoxicity of Magnetic Fields

**Figure 3:**
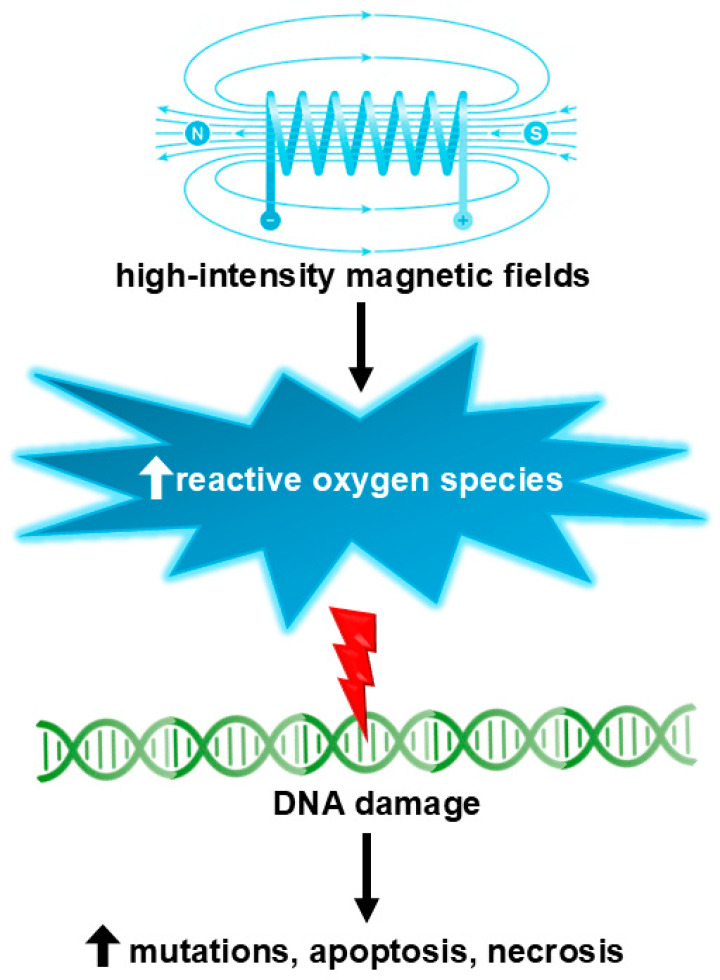
High-intensity (over 1 T) magnetic fields may induce genotoxic effects via the formation of reactive oxygen species.

It is widely acknowledged that SMFs below 1 T are not genotoxic [90,91]. Exposure to SMF has been shown to enhance the activity, concentration, and longevity of paramagnetic free radicals. Such an elevation in the generation of paramagnetic free radicals may potentially lead to oxidative stress, genetic mutations, necrosis, and/or apoptosis (Figure 3). The possible genotoxic effects resulting from exposure to SMF have been mainly investigated through studies conducted on cell cultures.

A study by Ohtani et al. evaluated whether intermediate-frequency magnetic fields (IF-MF) induce genotoxicity in murine models. The results indicated that there were no significant increases in either reticulocytes or mature erythrocytes in the group exposed to IF-MF compared to the control group. In germ cells, it was demonstrated that IF-MF exposure did not induce any genetic or chromosomal abnormalities. Consequently, based on these data, it can be concluded that IF-MF exposure did not exert any genotoxic effects on both somatic and germ cells [92].

Amara et al. conducted a study examining the impact of SMF exposure on DNA integrity within THP1 cells, a monocyte-derived cell line. In their experimental setup, cell culture flasks were subjected to SMF at a strength of 250 mT for durations of 1 h, 2 h, and 3 h. The findings indicated a marginal reduction in cell viability among the SMF-exposed groups in comparison to the control group. DNA integrity was assessed using single-cell gel electrophoresis, which demonstrated no significant DNA damage at 1 and 2 h of SMF exposure. Furthermore, the results indicated that an SMF exposure of 250 mT did not induce oxidative stress or DNA damage in the THP1 cells [93].

Potenza et al. analyzed the effects of SMF on cell proliferation and DNA integrity in human umbilical vein endothelial cells (HUVECs). The researchers discovered that a 4 h exposure of HUVECs to SMFs of IF-MF (300 mT) led to transient DNA damage at both nuclear and mitochondrial levels. This response was accompanied by an increase in mitochondrial DNA content and mitochondrial activity, as well as an elevated expression of several genes associated with mitochondrial biogenesis, observed 24 h after SMF exposure. After 48 and 72 h of exposure, no significant differences were observed between the exposed and control cultures. These findings imply that a 300 mT SMF does not induce permanent DNA damage in HUVECs and instead promotes a temporary increase in mitochondrial biogenesis [94].

Another study evaluated the impact of IF-MF on rat primary astrocytes, which were exposed for 24 h to 7.5 kHz MF at a magnetic flux density of 30 or 300 µT. The findings did not indicate any genotoxic or co-genotoxic effects of 7.5 kHz MFs at magnetic flux densities up to 300 µT, either in vitro or in vivo. Conversely, there was some indication that exposure to 7.5 kHz MFs might actually reduce the level of DNA damage. The most compelling evidence of any biological effects emerged from measurements of relative cell number, which was significantly and consistently elevated following MF exposure in all in vitro experiments [95].

Higher densities of SMF were investigated by Takashima et al. in DNA-repair defective mutants of *Drosophila melanogaster.* The authors discovered that exposing postreplication repair-deficient flies to MFs of 2, 5, or 14 T for a duration of 24 h resulted in a statistically significant increase in the frequency of somatic recombination [96]. Lee et al. have also taken into consideration high-field intensity MF. In this study, the genotoxic potential of 3 T clinical MRI scans on cultured human lymphocytes in vitro was examined through the analysis of chromosome aberrations, micronuclei, and single-cell gel electrophoresis. Following exposure to a 3 T MRI, there was a notable increase in the frequency of single-strand DNA breaks. These findings indicate that exposure to 3 T MRI can induce genotoxic effects in human lymphocytes [97].

Recently, Zafari et al. found that SMF reduces cisplatin resistance via increasing apoptosis pathways and genotoxicity in ovarian carcinoma cells. Following the treatment of various groups of cells for durations of 24, 48, and 96 h, the combined application of SMF and cisplatin resulted in a significant increase in DNA damage. This combined therapy notably elevated cellular mortality, predominantly through the induction of apoptosis, which was attributed to cell cycle inhibition. Furthermore, the co-treatment led to an upregulation in the expression levels of apoptotic genes, specifically P53 and P21. However, it did not substantially alter the expression levels of the BCL2 gene [98].

## 6. Neurological Effects

Ion flux across neural membranes enables neurons to propagate electric signals and leads to neuronal excitation [99]. This ion flux generates MF, which could be detected by, for example, magnetoencephalography methods [100]. Emerging evidence has shown a connection between external MF exposure and neural activity alterations such as neuron excitation [101]. For example, Ahmed and colleagues proved that PMF has an impact on the hippocampus, the brain region responsible for spatial orientation and memory acquisition. This impact results in long-term potentiation in hippocampus cells [102]. We described above the influences of GMF on biological systems. Wang and colleagues have shown an impact of GMF on neuronal activity. GMF stimulation results in collecting and processing magnetic receptors’ responses in the human brain [103]. As we mentioned above, MFs influence membrane protein activity and disrupt ion homeostasis, which alters membrane potential [104]. Hernando and colleagues created a model of brain stimulation using SMF. The authors estimated that tension in the cellular membrane affects ion channel activity, leads to an alteration in ion flux, and consequently impacts the cortex [105]. Prina-Mello et al. have shown that MF exposure increases intracellular Ca^2+^ levels in a neuronal cell line [106]. Rotem and colleagues found that a neural cell’s excitation, induced by MF, is dependent mostly on its coil orientation [107]. Aguila et al. proved that SMF could serve as a tool to modulate brain activity. The monkeys’ cortexes, after 0.5 T SMF exposure, were significantly more excited in comparison to the control, and the excitation was linked to animal behavior [108]. Christiansen et al. suggested that MF could modulate neuronal activity by modifying magnetosensitive ion channels using nanoparticles or gene modifications [109]. Modification of proteins to sense them for MF is referred to as “magnetogenetics”. This approach involved binding a ferromagnetic metal—i.e., iron—to channel protein. Thus, the modified channel’s opening and gating will depend on MF exposure [110].

## 7. Potential Therapeutic Applications of Magnetic Fields

### 7.1. Transcranial Magnetic Stimulation (TMS)

TMS is a non-invasive method of brain stimulation that applies a high (excitatory)- or low (inhibitory)-frequency magnetic field, which alters neuronal activity [111,112]. Repetitive TMS (rTMS) is used to induce changes in brain activity that can go beyond the stimulation period, while single-pulse TMS (including paired-pulse TMS) is typically used to investigate brain functioning [113]. Another variant of TMS is deep TMS (dTMS), which is similar to rTMS in general principle. To provide a deep focal point of stimulation, dTMS uses an H-shaped coil pattern, while rTMS requires a figure-of-eight coil [114].

TMS is approved by the FDA (Food and Drug Administration) for the treatment of resistant unipolar major depressive disorder (MDD), obsessive-compulsive disorder (OCD), smoking cessation, and comorbid anxiety in MDD [115,116,117,118]. Other possible indications for TMS include Parkinson’s disease, stroke rehabilitation, as well as neuropathic pain caused by peripheral nerve disorders, fibromyalgia, and migraine [119,120,121,122,123]. Regarding epilepsy, a consensus for TMS has not been established, yet TMS can trigger seizures, although some authors highlight that TMS may be beneficial and the overall risk is low (less than 1%) [124,125,126]. However, further clinical trials should be conducted to reach a definitive conclusion.

### 7.2. Magnetic Seizure Therapy (MST)

Electroconvulsive therapy (ECT) is an effective procedure widely used for depression and other major psychiatric disorders [127]. Despite its effectiveness, ECT may cause serious or long-lasting adverse effects, e.g., retrograde amnesia or severe cardiovascular, pulmonary, and cerebrovascular events [128]. Magnetic seizure therapy (MST) involves inducing therapeutic seizures with high-frequency rTMS [129]. MST is a possible superior alternative to ECT since MST may not negatively affect cognitive functions. In MST, magnetic fields are produced using a modified TMS device, which has a propensity to generate a higher output than a conventional TMS device [130].

MST is effective in the treatment of MDD and suicidality in patients with resistant depression [131,132,133]. MST is performed under general anesthesia (e.g., methohexital, remifentanyl, or ketamine) with a muscle relaxant (e.g., succinylcholine) [133]. Thus, possible mild drug complications, such as sore throat, dysphagia, nausea, vomiting, pain, and swelling, should be acknowledged [134]. Another potential adverse effect related to the clicking noise of the MST magnetic coil is hearing loss, which should be prevented by wearing earplugs [135]. A clinical trial (NCT03191058) is currently being conducted to assess the efficacy, tolerability, and cognitive adverse effects of MST [136].

## 8. Electromagnetic Sensing and Imaging

### 8.1. Magnetocardiography

Magnetocardiography (MCG) is a non-invasive technique that measures magnetic fields generated by the electrical conduction system of the heart. In contrast to an electrocardiogram (ECG), the magnetic fields in the MCG are not attenuated by variations in tissue conductivity [137]. Furthermore, MCG does not require electrode placement on the skin. Instead, highly sensitive magnetic sensors are located outside the body. Research suggests that the combination of ECG and MCG may increase sensitivity to the identification of myocardial infarction [138,139]. MCG was effective in detecting ischemia in patients with normal ECG and negative cardiac biomarkers [140]. Recently, the application of MCG in the detection of inflammatory cardiomyopathy and monitoring immunosuppressive therapy was evaluated. The results of the study were promising; MCG revealed a response to treatment after 7 days, while changes in echocardiography were observed after 30 days [141]. Another possible indication for MCG is coronary artery disease (CAD). Chaikovsky et al. obtained a sensitivity of 93.8% and a specificity of 87.1% in CAD [142]. Due to its safety, MCG is also a diagnostic option for fetal arrhythmias [143,144].

### 8.2. Magnetoencaphalopathy

Similarly to MCG, magnetoencaphalopathy (MEG) is a non-invasive procedure that detects weak magnetic fields generated by neuronal activity in the brain. Currently, MEG is performed in equivocal cases prior to epilepsy surgery and functional brain activity mapping [145,146]. Brain activity mapping can be performed simultaneously with EEG (electroencephalography) and MEG. Both modalities have complementary spatial sensitivity profiles—EEG has higher sensitivity for deeper sources, while MEG visualizes better superficial areas. The MEG-evoked responses during neurological examination are accurate in finding Wernicke and Broca areas in patients with aphasia [147].

### 8.3. Magnetic Resonance Imaging (MRI) Enhancement

Implementing the magnetic resonance imaging (MRI) technique was undoubtedly an enormous milestone for clinical medicine. MRI is considered a gold standard for the diagnosis and monitoring of various soft tissue-associated diseases, as well as disorders of the nervous and musculoskeletal systems [148,149,150]. MRI scans are high resolution, especially after the administration of gadolinium-based contrast agents (GCAs).

GCAs are paramagnetics; hence, they lose magnetization when the magnetic field is removed [151]. GCAs enable both longitudinal and transverse magnetic relaxation and enhance the signals of T1, mainly by shortening T1 more than T2. GCA agents are generally safe, although they may trigger serious, rare adverse events, including systemic nephrogenic fibrosis and acute respiratory distress syndrome [152,153].

## 9. Biomedical Applications of Magnetic Fields

### 9.1. Magnetic Hyperthermia

The heating potential of magnetic nanoparticles is a crucial feature that enables the application of moderate hyperthermia (temperatures over 41 °C; up to 46 °C) and magnetic thermoablation (temperatures over 46 °C; up to 56 °C) [154]. Increased temperature implicates numerous effects at the cellular level. First, heating raises the metabolism rate and accelerates biochemical reactions consistent with the van Hoff equation. Second, structural intracellular transformations of DNA, RNA, and proteins may cause damage to cellular membranes and the cytoskeletal system. As a result, increased cellular permeability may facilitate drug delivery [155]. Third, the expression of heat shock proteins (HSP) may be upregulated [156]. Additionally, heating may trigger alterations in the tumor microenvironment (TME). Heating may affect TME by inducing hypoxia, pH changes, perfusion, and immunological system dysregulation [154,157].

### 9.2. Magnetic Targeting

Currently, personalized therapy based on the upregulation or downregulation of specific proteins is the goal of managing several cancers. Legge et al. investigated in vitro whether magnetic iron oxide nanoparticles may be effective in oral squamous cell carcinoma (SCC) [158]. Due to the upregulation of αvβ6 integrin in SCC, targeted therapy with magnetic thermoablation of tumor cells is a promising option to be further studied [159]. In another in vitro study, magnetic nanoparticles were conjugated with sortilin, a human IgG1 monoclonal antibody against ovarian cancer cells. Subsequently, calcium hydroxide and Taxotere were administered. The results of the study were remarkable since the tumor growth inhibition rate was estimated at 78% [160].

### 9.3. Regenerative Medicine

Emerging evidence points out that MF is a potential tool in regenerative medicine and tissue engineering, such as osteoporosis treatment. Pi and colleagues found that PEMF (75 Hz, 1 mT) inhibits osteoclastic differentiation but does not affect cell viability, which is important for proper bone mass maintenance [161]. Interestingly, Bekhite et al. found that EMF (0.4–2 mT) promotes embryonic cell osteogenesis and vasculogenesis in mice. These effects were probably driven by increased levels of ROS [162]. Pesqueira and colleagues suggested LF-SMF as a potential tool to regenerate tendons. Their research delivered evidence that MF results in the inhibition of pro-inflammatory interleukin secretion, upregulation of collagen genes, and enhancement of anti-inflammatory interleukin secretion [163].

**Figure 4:**
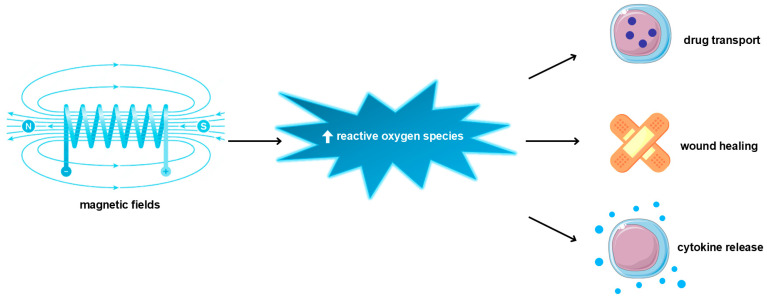
Biomedical applications of magnetic fields are mediated by reactive oxygen species upregulation.

There is proof that MF’s impact on tissue promotes healing processes, especially wound healing. In wound closure, various components are involved, for example, epithelial cells, connective tissue cells, and immune cells, which promote collagen production, matrix metalloproteinase activity, growth factor release (e.g., VEGF, FGF, PDGF, TNF, HGF, and IL-1), and inflammatory environment progression [164]. The inflammatory state is an essential step in wound healing due to immune cell recruitment, leading to microbial elimination and fibrosis promotion [165]. However, non-healing wounds—pathological states—are characterized by chronic inflammation—the existence of pro-inflammatory cytokines or growth factors, like TNF-1α, IL-1β, TGF-β, and pro-inflammatory immune cells (macrophages or neutrophiles) [164,166,167]. Additionally, in this pathological state, tissue remodeling is inhibited by MMP overproduction [168]. Many disorders are struggling with non-healing wounds, for example, diabetes [169]. Martino et al. showed that endothelial cells’ exposure to a pulsed electromagnetic field (PEMF) in a ratio of 60 μT increased their proliferation rate. Additionally, PEMF (120 μT) promoted overexpression of endothelial nitric oxide synthetase, the enzyme related to ROS production [170]. ROS overproduction is a hallmark of various diseases but also participates in wound healing as a secondary messenger and promotes wound closure through, i.e., angiogenesis mediation [171]. There is experimental proof that a weak magnetic field alters ROS production [172] (Figure 4). The influence of MF on ROS could be twofold. Evidence found by Wang and colleagues suggests that, in many cases, MF increases ROS release [173]. The research by Poniedziałek and colleagues delivers proof that short exposure of SMF (max = 60 mT) causes inheritance in ROS production, while longer exposure reverses this effect [174]. In Martino’s research, the reduction in MF from the GMF range of 0.2–2 μT resulted in suppressing ROS production in HT1080 and AsPC-1 cell lines [170]. Castello and colleagues postulated that weak frequencies (5–10 MHz) of MF could inhibit a cell’s growth rate by promoting ROS production [175]. Gurhan and colleagues found that SMF and radiofrequency fields (1.8–7.2 MHz) decrease ROS release and interior pH in the HT 1080-fibrosarcoma cell line [176]. However, Romeo et al. did not detect any alteration in ROS production in human fetal lung fibroblasts by SMF (350 mT) treatment [177]. Moreover, MF also has an impact on in vivo cell growth rate, regeneration, and thus wound healing. Van Huizen and colleagues used the planarian regeneration model to check it. The authors observed stem cell alteration in proliferation rate and linked it with ROS upregulation and stress response protein overexpression, like Hsp70 [172]. MF, via promotion mechanisms involved in the regeneration process, accelerates the wound healing process. Saliev and colleagues observed enhanced MMP-9 activity and also downregulation of pro-inflammatory cytokine secretion in keratinocyte cell lines as a result of ELF-SMF exposure [177]. MF promotes the skin wound healing process [178]. Delivered data showed a shortened time of wound closure in rats as a result of low-power SMF exposure [179]. MF reduces the expression of oxidative stress markers, like NRF2, which participate in ROS release and the oxidative stress response. Thus, it decreases ROS levels and enhances cell viability and migration to the wound environment [180]. Also, clinical trials confirm MF’s effectiveness in wound healing processes and diabetic wounds [181].

As Wu and colleagues noticed, MF has an impact on MSC physiology [15]. MSCs are a subtype of multipotent stem cells, which, besides their differentiation ability, are much easier to isolate and culture in vitro [80]. Additionally, MSCs are distinguished by immunomodulatory properties, such as anti-inflammatory cytokines and growth factor release, and thus inhibit the immune response to inflammation tissue [182]. Accumulated data suggest that MF promotes mesenchymal stem cell proliferation, which indicates that is a potential approach to regenerative medicine and cell-based therapies [182]. One of the therapeutic advantages of MSCs application is their migration ability to inflammation tissue [183].

## 10. Interactions with Nanoparticles

Nanoparticles have emerged as promising agents in various biomedical applications due to their unique physical and chemical properties. Among these, magnetic nanoparticles (MNPs) have garnered considerable interest owing to their responsiveness to external magnetic fields [184].

The interaction between MFs and nanoparticles is governed by several physical phenomena, including magnetic susceptibility, magnetization, and Brownian relaxation [185]. MNPs possess magnetic moments that align with an external MF, leading to the generation of magnetic forces. This alignment induces a torque on the MNPs, causing them to reorient and migrate towards regions of higher MF intensity. Additionally, alternating MFs can induce oscillatory motion in MNPs, resulting in heat generation through mechanisms such as Neel and Brownian relaxation [185].

Cancer hyperthermia using MNPs has emerged as a promising therapeutic approach for localized tumor treatment [186]. By exposing tumor tissues to alternating MFs, MNPs can generate heat, leading to localized hyperthermia and tumor cell death. Kobayashi and Dürr et al. have demonstrated the efficacy of MNP-mediated hyperthermia in preclinical and clinical settings, highlighting its potential as a minimally invasive cancer treatment modality [186,187].

MF interactions with nanoparticles offer precise control over drug delivery and release kinetics. Liu et al. proposed the use of MNPs as carriers for therapeutic agents, where drug-loaded MNPs can be guided to the target site using external magnetic fields. Upon reaching the desired location, the application of magnetic fields can trigger the release of the encapsulated drug, enhancing its local concentration and therapeutic efficacy [155].

MNPs have also found applications in diagnostic imaging and sensing. Tao et al. demonstrated the use of MNPs for efficient viral RNA extraction and detection, leveraging their magnetic properties to isolate target molecules from complex biological samples. Additionally, MNPs functionalized with targeting ligands can be employed for magnetic resonance imaging (MRI) and magnetic particle imaging (MPI), enabling high-resolution imaging of diseased tissues [188].

MNPs exhibit unique physical, chemical, structural, and magnetic properties, enabling selective attachment, manipulation, or transport under external magnetic fields. Beyond serving as components of drug delivery systems or contrast agents, nanoparticles can act independently as treatment agents, such as intracellular hyperthermia inducers [184]. Despite the promising potential of magnetic field interactions with nanoparticles in biomedical applications, several challenges need to be addressed. These include optimizing MNP properties for enhanced magnetic responsiveness, improving targeting specificity, and minimizing off-target effects [189]. Furthermore, the translation of MNP-based therapies from preclinical studies to clinical practice requires rigorous evaluation of safety, efficacy, and scalability. Understanding the diverse characteristics of magnetic nanoparticles and their formulations in biological systems remains a significant challenge for nanoscience in the coming years.

## 11. Conclusions

In summary, the investigation into the cellular and molecular impacts of MFs has yielded substantial insights into their interactions with biological systems. In this article, we have explored the diverse ways in which MFs affect cellular structures, signaling pathways, DNA integrity, gene expression, protein function, enzyme activity, cellular stress responses, proliferation, and apoptosis. Furthermore, the potential therapeutic applications and safety concerns of MF exposure have been examined, underscoring both the opportunities and the challenges associated with utilizing MFs for medical and biotechnological purposes.

MFs can modify cellular morphology and function by interacting with membranes, organelles, and the cytoskeleton. These interactions are based on fundamental physical principles governing electromagnetic phenomena. MFs influence ion channel activity, receptor functions, and intracellular signaling pathways, leading to alterations in cellular communication and response mechanisms. MFs affect protein conformation and enzyme activity, thereby influencing various biochemical pathways crucial for cellular function and homeostasis.

MF exposure impacts DNA structure, replication, and repair processes, with potential implications for gene expression and mutagenesis. These effects emphasize the necessity of understanding MF interactions at the genetic level. Cells exhibit stress responses to MF exposure, such as the activation of heat shock proteins and oxidative stress pathways. Adaptive mechanisms may develop with prolonged exposure, reflecting the dynamic nature of cellular responses. MFs can either stimulate or inhibit cellular proliferation and apoptosis, depending on the context and exposure conditions. This duality presents both opportunities and risks that require careful consideration. It is widely recognized that SMF with an intensity below 1 T does not exhibit genotoxic effects. However, there are reports indicating that higher-intensity MFs, e.g., those utilized in 3 T MRI scans, can induce genotoxic effects.

MFs show promise for therapeutic applications in areas such as wound healing, pain management, and cancer treatment. Ongoing clinical studies are exploring these possibilities, supported by an expanding body of experimental evidence. Understanding the health implications of MF exposure is crucial, given the widespread presence of MFs in occupational and environmental settings. Regulatory standards are needed to mitigate potential risks and protect public health.

## 12. Future Directions and Challenges

MF could serve as a tool to manipulate cell fate using MNPs [190]. The manipulation requires MNPs targeted to essential proteins involved in key cellular signaling pathways, like cell proliferation, differentiation, cell-to-cell communication, and cell death. Additionally, MNPs can lead to structural alterations of targeted proteins [191]. For example, Bharde and colleagues delivered proof of the ability of designed supermagnetic iron oxide MNPs to bind with EGFR and thus activate it in the presence of strong MF. Interestingly, magnetosensitive activation results also exist in downstream signaling pathways in which EGFR is involved [192]. These signaling pathways direct many physiological processes, like cell viability, proliferation, apoptosis, growth, and cycle progression; taking control of them could serve as a treatment approach for diseases associated with cell over-proliferation [193].

Additionally, MNPs could serve as “molecular buttons” to turn on/off cell death. Cho and colleagues designed MNPs that are targeted and bind to death receptor 4 (DR4), the protein that participates in apoptotic pathways. MF exposure (0.2 and 0.5 T) resulted in DR4-mediated apoptotic pathway activation in colon cancer cell lines as well as in vivo models of zebrafish [194]. Importantly, MNPs could be co-loaded with drugs and serve as targeted treatments, for example, in cancer diseases. Wu et al. created MNPs co-loaded with tetrandrine, a chemotherapeutic drug. The conjugate displays significant cytotoxic properties in the in vitro lung cancer cell line model [195].

## Figures and Tables

**Table 1:** Summary of thresholds for the effects of magnetic fields.

Magnetic Field Parameters	Cell Line	Biological Effects	Ref.
50 Hz, 1 mT for 1 h	HaCaT keratinocyte	Induced proliferation via mTOR (PI3K/Akt) and ERK signaling pathways	[2]
60 Hz, 1.5 mT for 144 h	HaCaT keratinocyte	Inhibited cell growth, activated ATM-Chk2-p21 pathway (G1 arrest)	[3]
60 Hz, 6 mT for 30 min every 24 h for 3 days	IMR90, HeLa	Induced DNA double-strand breaks (DSBs) and apoptosis via p38 activation; increased micronucleus formation and chromosomal aberrations.	[4]
60 Hz, 7 mT for 10–60 min	IMR90, HeLa	Induced DNA DSBs without apoptosis, activated DNA damage checkpoint without ROS production; increased DNA repair foci formation	[5]
50 Hz, 1 mT for 24/48/72 h	SH-SY5Y	Significantly increased ROS levels	[6]
2 ± 0.2 mT; 75 ± 2 Hz for 10 min, 4 times/week	SH-SY5Y	Decreased H2O2-induced ROS	[7]
30 Hz, 0.8 mT for 1 h	6B 1 hybridoma	Inhibited proliferation; variable effects at different frequencies	[8]
60 Hz, 20, and 200 μT for various times up to 30 h	Human fibroblasts	Increased G1 phase length at lower intensities, no effect at higher flux densities	[9]
50 Hz, 100 μT, 0.6 mT, 24 h, 48 h	K562, DU145 cells	Significantly increased cell proliferation; micronucleus formation observed	[10,11]
50, 60 Hz, 2, 20, 100, 500 μT, 24 h, 48 h	K562 cells	No effect on cell proliferation	[12]
(104–105) Tm−1, static	Cancer cells enriched by Fe	Tumor arrest	[13]
(103–105) Tm−1, static	HeLa cells, other cancerous cells with low membrane tension	Magnetically assisted cell division	[13]
(102–103) Tm−1, static	PC-3 cells and fibroblasts	Magnetically assisted endocytosis	[14]
103 Tm−1, static	THP-1 cells	Cell swelling	[15]
50 Hz, 1 mT for 12 h/day for 3 days	Human lymphocytes	Increased ROS production and DNA fragmentation; increased micronucleus frequency and chromosomal aberrations	[16]

ROS—reactive oxygen species; HaCaT—human epidermal keratinocyte cell line; IMR90—human lung fibroblast cell line; HeLa—human cervical carcinoma cell line; SH-SY5Y—human neuroblastoma cell line; PC-3—human prostate cancer cell line; K562—human chronic myelogenous leukemia cell line; THP-1—human acute monocytic leukemia cell line.

## Data Availability

Data sharing is not applicable as no datasets were generated or analyzed during the current study.
